# Validation of the Friedewald Formula in Patients with Metabolic Syndrome

**DOI:** 10.1155/2014/261878

**Published:** 2014-02-06

**Authors:** José Knopfholz, Caio César Diniz Disserol, Andressa Jardim Pierin, Fernanda Letícia Schirr, Larissa Streisky, Lilian Lumi Takito, Patrícia Massucheto Ledesma, José Rocha Faria-Neto, Marcia Olandoski, Claudio Leinig Pereira da Cunha, Antonio Milton Bandeira

**Affiliations:** Pontifícia Universidade Católica do Paraná, Rua Imaculada Conceição, Prado Velho, 1155 Curitiba, PR, Brazil

## Abstract

Currently, the Friedewald formula (FF) is the main method for evaluating low-density lipoprotein cholesterol (LDL-c). Recently, many limitations have emerged regarding its use, including patients with triglyceride levels ≥400 mg/dL, diabetes mellitus, and kidney or hepatic chronic diseases. We analyzed the use of the FF in patients with metabolic syndrome. We selected patients with known metabolic syndrome that fulfilled the *National Cholesterol Education Program Expert Panel on Detection, Evaluation and Treatment of High Blood Cholesterol in Adults (Adult Treatment Panel III) Final Report* and excluded patients with triglyceride levels ≥400 mg/dL and chronic liver and/or kidney disease. Using direct assays, we measured total cholesterol, high-density lipoprotein cholesterol, triglycerides, and LDL-c. Then, LDL-c was estimated using the FF and compared with the LDL-c by direct assay. The sample size was 135 patients. Using the FF, the mean LDL-c value was 124.4 ± 42.1 mg/dL; it was 125.1 ± 38.5 mg/dL by direct assay. The correlation coefficient between these two methods was 0.89, with statistical significance (*P*  value < 0.001). There were no significant differences between the patients with triglyceride levels >150 mg/dL (*P* = 0.618). In conclusion, FF is a good method for estimating LDL-c in patients with metabolic syndrome.

## 1. Introduction

Metabolic syndrome (MS) comprises a group of metabolic abnormalities that are related to high cardiovascular risk, particularly for the development of coronary artery disease (CAD) [1].

MS is highly prevalent in Brazil, affecting approximately 30% of the population. The prevalence increases in older populations [2].

The main concern about MS is the development of CAD, which is a highly prevalent condition and a major cause of mortality. In the development of CAD, lipid metabolism, which is the formation of atherosclerotic plaque, plays a major role. Hypercholesterolemia is a lipid abnormality commonly related to atherosclerosis. Nevertheless, LDL-c, which is the major lipoprotein associated with CAD, is not a part of the diagnostic criteria of MS [3–5]. The physiological levels of LDL-c that are sufficient for lipid metabolism range from 25 to 60 mg/dL, and LDL-c is more atherogenic when it exceeds 100 mg/dL. Therefore, as previously described in the literature, lower levels of LDL-c reduce cardiovascular morbidity and mortality [6–8].

Cardiovascular risk stratification defines the LDL-c value target. Therefore, the LDL-c measurement technique requires standardization and good accuracy [6–10].

The Friedewald formula (FF) is an estimation of LDL-c level that uses the following levels of total cholesterol (TC), triglycerides (TG), and high-density lipoprotein cholesterol (HDL-c): LDL-c (mg/dL) = TC (mg/dL) − HDL-c (mg/dL) − TG (mg/dL)/5 [6, 11–13]. To be applied in the FF, the measurements of TC, HDL-c, and TG must be in mg/dL; the estimation differs and was not performed for the mmol/L measurements. The FF became the standard method for LDL-c assessment because it is economical and simpler than direct assays, the most accurate LDL-c measurement methods [9, 11–13].

FF has limitations under certain conditions, primarily when metabolic abnormalities alter the relationship between very-low-density lipoprotein cholesterol (VLDL-c) and TG, as in high hypertriglyceridemia (TG > 400 mg/dL) [11, 13–16]. Furthermore, new studies show considerable differences between the estimation and direct assessment of LDL-c in many other conditions [6, 7, 12, 13].

As in MS, there are changes in the disposition and metabolism of lipids; thus, the FF estimates LDL-c by assessing other lipid particles. Likely, its use in MS may not be reliable.

## 2. Materials and Methods

We selected patients with known metabolic syndrome that fulfilled the criteria outlined in the *National Cholesterol Education Program Expert Panel on Detection, Evaluation and Treatment of High Blood Cholesterol in Adults (Adult Treatment Panel III) Final Report* [6, 12]; three or more of the following components were present: increased waist circumference (≥102 cm for men and ≥88 cm  for women); triglycerides ≥ 150 mg/dL or drug treatment for elevated TG; low HDL-c (<40 mg/dL for men and <50 mg/dL for women) or drug treatment for low HDL-c; systolic blood pressure ≥ 130 mmHg, diastolic blood pressure ≥ 85 mmHg, or treatment with antihypertensive in patients with a history of hypertension; fasting glucose ≥ 100 mg/dL or treatment for high blood glucose.

Patients who fulfilled the MS criteria, consented to provide a blood sample, and signed the informed consent form were included in the study. Patients who did not fulfill the MS criteria, did not sign the informed consent form, and had TG ≥ 400 mg/dL were excluded.

All participants underwent a 12-hour fast. The following tests were performed (using a Selectra II analyzer with reagents and calibrators from ELITech): direct assays for TC, HDL-c, LDL-c, and TG. The results were applied in the FF, and then the LDL-c estimation could be performed. LDL-c was determined by a homogenous direct assay (i.e., colorimetry) using an ELITech kit. Colorimetry is a third generation method (a homogeneous assay with some reagents that can solubilize or specifically block these lipoproteins, dosing LDL-c alone in the same bucket with an enzymatic reaction) [17]. Thus, we could compare both LDL-c values (using the FF and by direct assay) and evaluate the reliability of the FF in the MS patients.

The results were described as means, medians, minimum values, maximum values, and standard deviations (quantitative variables) or by frequency and percentiles (qualitative variables). For the assessment of the results of LDL-c using the FF and LDL-c by direct assay was used the Student's *t*-test for paired samples. To evaluate the correlation between both methods, Pearson's correlation coefficient was used. Scattergram data and a Bland-Altman diagram were used to evaluate the dispersion and differences between the results obtained using the FF and direct assay, and *P* values < 0.05 were considered to be statistically significant. Data were analyzed with the software Statistica v.8.0.

## 3. Results

**Figure 1:**
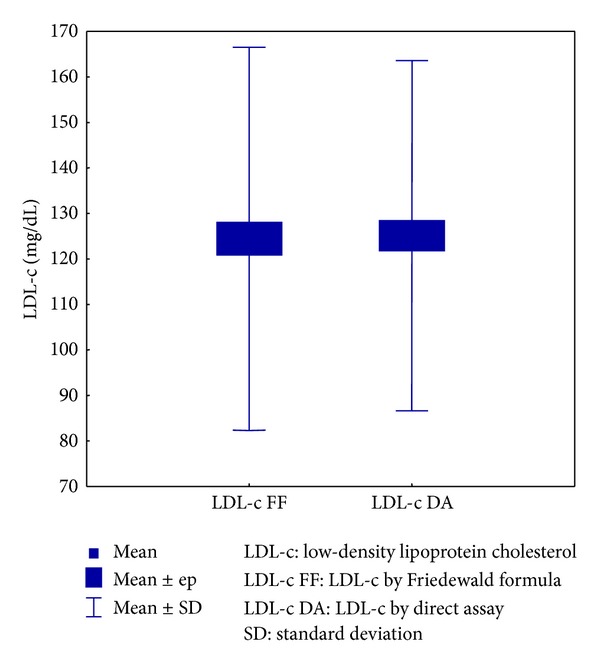
Relationship between the LDL-c values using the FF and direct dosage.

The sample size comprised 135 individuals. Using the FF, the statistical analysis of LDL-c showed a mean value of 124.4 mg/dL (SD = 42.1 mg/dL); by direct assay, the mean value was 125.1 mg/dL (SD = 38.5 mg/dL). The difference between the FF and direct measurement showed strong correlation between both methods because the mean difference was −0.7 mg/dL, as shown in Figure 1.

**Figure 2:**
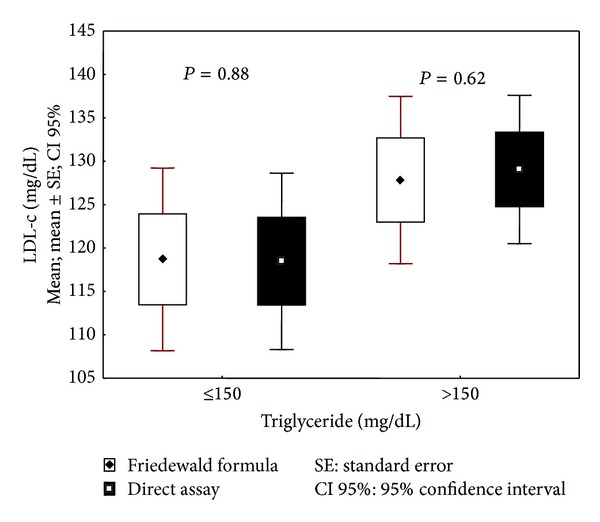
Evaluation of LDL-c values by triglyceride level with the FF and direct assay.

We subdivided the patients based on their TG values to analyze whether the different methods used produced different values for this lipid. In the group of patients with TG ≤ 150 mg/dL (*n* = 50), no significant difference between the methods (*P* = 0.881) was observed; in the patient group with TG > 150 mg/dL, there was also no significant difference between the two methods (*P* = 0.618), as shown in Figure 2.

**Figure 3:**
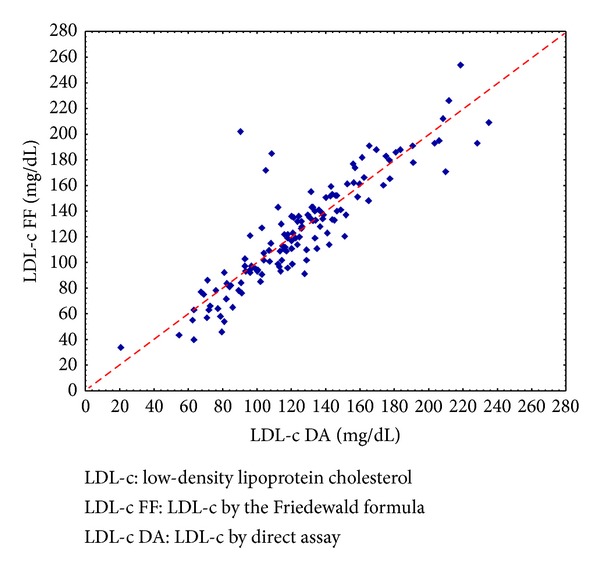
Scattergram data between LDL-c values by Friedewald formula and by direct assay.

To assess the degree of association between the methods, we estimated the correlation coefficient between them, which equaled 0.89, with statistical significance (*P* < 0.001). Thus, based on the results of the statistical tests, we believe that there is no significant difference between the assessment of LDL-c using the FF and by direct measurement, as shown in Figure 3.

**Figure 4:**
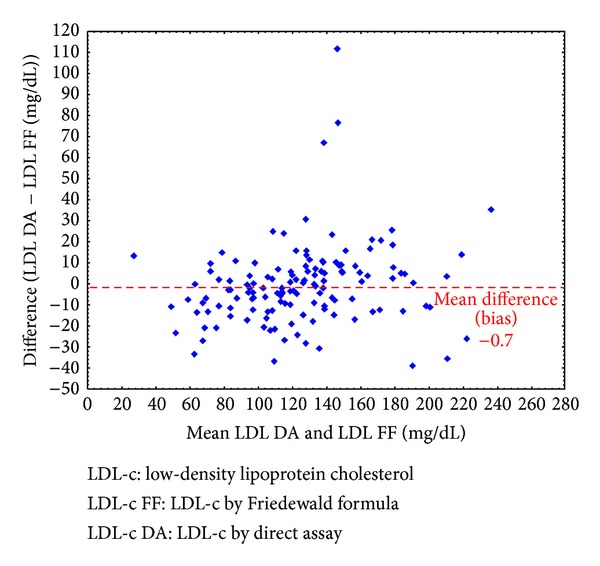
A Bland-Altman diagram correlating the absolute difference between the two methods and their means.

As shown in Figure 4, it is possible to conclude that, in general, the FF underestimates the value of LDL-c compared to direct measurement. Moreover, the average difference between these methods appears to be more pronounced when the LDL-c is lower (by direct measurement): when LDL-c ≤ 121 mg/dL, the mean difference was 0.26 mg/dL, and for LDL-c > 121 mg/dL, the mean difference was −1.62 mg/dL. However, despite being approximately six times greater when the absolute difference in LDL-c > 121 mg/dL, this result is still too small and is clinically insignificant.

A minority of patients (*n* = 9) demonstrated an absolute difference >30 mg/dL between both methods, which could be clinically significant, as shown in Figure 4. Eight of these patients had TG > 150 mg/dL, but just one patient had a level >300 mg/dL. Considering that the mean value of TG in all patients with TG > 150 mg/dL in this study was 219.1 mg/dL, we concluded that patients with important differences in their LDL-c values (using the FF and direct assay) were not clustered in higher triglyceride level group.

The relative difference between the calculated value of LDL-c using the FF and the direct measurement was that, on average, the value of the FF is 0.28% lower than the direct measurement.

## 4. Discussion

As the relationship between serum LDL-c and cardiovascular disease is well established, reliable methods of measuring this lipid are needed both to classify it and to treat our patients. However, recently, many studies have demonstrated limitations to the most widely used method for serum LDL-c estimation, the FF.

Despite the classical indication for direct measurement of LDL-c as TG > 400 mg/dL, some studies have indicated that, for lower TG values, the FF is not as reliable. From 180 mg/dL, the FF already shows significant differences (overestimating LDL-c values) when compared to direct measurement methods [17, 18]. Similar results were shown by Charuruks and Milintagas [19], who indicated the direct measurement of LDL-c when TG ≥ 200 mg/dL because they found that the direct method was more precise and accurate than FF, even for TG levels between 200 and 399 mg/dL. One Brazilian study showed a similar conclusion for FF use [20]. Nevertheless, these findings do not align with those of our study, in which the LDL-c value was estimated with precision by the FF for any value of TG < 400 mg/dL.

Some studies have shown that FF can also display discrepancies in low TG values [17, 21, 22]. When TG value was <70 mg/dL, the estimated LDL-c using the FF showed slightly lower values than that using the direct method [17]. Contradictory results have been demonstrated in other studies, in which serum LDL-c using the FF was higher than the homogeneous assay for TG < 100 or 200 mg/dL [21, 22]. In the present study, LDL-c values using the FF were virtually identical to the values by direct assay, particularly when TG ≤ 150 mg/dL (mean difference between LDL-c using the FF and direct measurement = 0.2).

New studies are demonstrating the limited efficacy of FF in diabetic patients. Diabetes is the epitome of MS; thus, many of these studies have affirmed that this estimation is not as accurate for this syndrome as previously believed [6, 18, 23]. In diabetic patients, with or without insulin use, the FF underestimates, on average, 8% serum LDL-c, but it can underestimate more than 10% in patients with TG levels between 200 and 400 mg/dL [18].

We found only one study in the literature that correlated the efficacy of FF and a direct assay specifically in patients with MS. The authors found that the direct measurement method is more accurate than the FF in these patients; these results represent the limitations of this indirect method. However, the authors also noted that even direct assays have limitations in identifying small and dense LDL-c, which is abundant in these patients [23]. In the current study, all patients with MS and TG < 400 mg/dL demonstrated reliable LDL-c value estimates using the FF.

## 5. Conclusion

In conclusion, FF is a reliable method to estimate serum LDL-c in patients with MS.
